# Protective effect of resveratrol on retinal damage in glaucoma: a systematic review and meta-analysis of preclinical studies

**DOI:** 10.3389/fphar.2024.1521188

**Published:** 2025-01-15

**Authors:** Feng Zhang, Tao Li, Junli Wan, Lu Wang, Wenmei Guo, Yue Hu, Hao Wang, Wei Bian

**Affiliations:** ^1^ Southwest Hospital/Southwest Eye Hospital, Third Military Medical University (Army Medical University), Chongqing, China; ^2^ Key Lab of Visual Damage and Regeneration and Restoration of Chongqing, Chongqing, China; ^3^ School of Acupuncture and Tuina, Chengdu University of Traditional Chinese Medicine, Chengdu, China

**Keywords:** resveratrol, glaucoma, retina, preclinical studies, systematic review and meta-analysis

## Abstract

**Introduction:**

Resveratrol, a polyphenolic compound commonly found in natural plants and fruits, exhibits potential in preventing optic nerve damage in glaucoma, as indicated by several animal studies. However, there is presently a dearth of relevant evidence available for comprehensive summarization.

**Methods:**

In this study, we conducted an extensive search across 7 electronic databases, encompassing all pertinent animal studies for a systematic review and meta-analysis. Methodological quality was evaluated using SYRCLE’s bias risk tool, with statistical analysis performed using Stata 17.0. The primary outcome measures included the survival of retinal ganglion cells and retinal thickness.

**Results:**

The comprehensive analysis of the 30 included studies revealed that resveratrol can enhance the expression of Sirtuin 1(SIRT1) protein in retinal tissue (SMD: 3.00, 95% CI: 2.46, 3.53, P = 0.095), boost the survival rate of retinal ganglion cells (SMD: 4.33, 95% CI: 3.28, 5.38, P < 0.05), decelerate the thinning of retinal thickness (SMD: 4.26, 95% CI: 2.77, 5.75, P < 0.05), and enhance visual function. Its potential mechanism of action may involve the suppression of pro-inflammatory cytokine levels and cell apoptosis.

**Discussion:**

Resveratrol emerges as a promising agent for mitigating glaucoma-related retinal damage. However, given that the animal research models utilized in the study may not fully reflect the intricate scenarios of multiple coexisting diseases in clinical settings, and the administration methods in animal models may differ from those in clinical practice, future studies should aim to provide higher levels of evidence to facilitate the clinical translation of these findings.

**Systematic Review Registration:**

identifier [CRD42024535673].

## 1 Introduction

Glaucoma is an ocular condition characterized by the pathological elevation of intraocular pressure, leading to mechanical compression, optic nerve ischemia, and subsequent optic nerve damage along with visual field defects. With a blindness rate ranging from approximately 5%–20%, glaucoma stands as the second most prevalent cause of blindness in ophthalmology, following cataracts (Bourne et al., 2018). Research data indicates that a minimum of 80 million individuals globally are afflicted by glaucoma, and projections suggest that the number of patients is poised to surpass 110 million by the year 2040 (Liu et al., 2023; Tham et al., 2014). A healthcare cost research report revealed that glaucoma had a heavy disease burden (Prager et al., 2016). Glaucoma has imposed a substantial economic burden of $2.9 billion on the US economy (Thomas et al., 2015), highlighting its significance as a medical burden not only within the US but also globally (Barayev et al., 2021; Patel et al., 2021). Due to its high incidence rate, elevated blindness prevalence, and substantial economic impact, glaucoma has emerged as a noteworthy public health concern deserving attention.

The pathogenesis of glaucoma is intricate, with a prevailing belief that alterations in mechanical pressure and disruptions in blood flow regulation play significant roles (Flammer et al., 2002; Baudouin et al., 2021). Increased intraocular pressure or other vascular risk factors that potentially decrease ocular blood flow can result in inadequate blood delivery to the optic nerve. The cascade of ischemia and hypoxia can induce harm to retinal neurons, particularly axonal degeneration and diminished retinal ganglion cell (RGC) counts, culminating in irreversible impairment of visual function (Li et al., 2018; Liu et al., 2019). The activation of inflammatory responses and cell apoptosis within retinal tissue is intricately linked to RGC damage. In the compromised retina, the interplay of inflammatory cytokines such as iNOS (Liu and Neufeld, 2003; Shareef et al., 1999), COX-2 (Deng et al., 2020), IL-6 (Hu et al., 2021), and IL-1β (Chen et al., 2020; Coyle et al., 2021) instigates the immune-inflammatory response in the glaucomatous retina and triggers apoptosis of RGC cells. Studies indicate that in a mouse model of acute intraocular hypertension, retinal nerve cells undergo progressive apoptosis (Zhou et al., 2019), with aberrant expression levels of caspase-3, Bax, and Bcl-2 potentially serving as pivotal factors in RGC apoptosis (Quigley et al., 1995; Risner et al., 2022; Zalewska et al., 2004; Phatak et al., 2016). Among the various risk factors contributing to glaucoma damage, elevated intraocular pressure stands out as the modifiable factor (Fernandez-Albarral et al., 2024). As a result, the primary approach to treating glaucoma involves medication or surgical interventions aimed at lowering intraocular pressure. Nevertheless, the reduction of intraocular pressure alone may not entirely halt the progression of glaucoma or prevent damage to RGCs (Zeng et al., 2023). Retinal ischemia-reperfusion injury could be a crucial factor influencing treatment efficacy (Flammer et al., 2002; Goldblum and Mittag, 2002). Under the combined influence of various pathological mechanisms during ischemia-reperfusion, RGCs may experience cell death, morphological degeneration, and functional loss, ultimately resulting in vision impairment (Kim et al., 2013). Given the complexity of the underlying mechanisms, effective treatment methods are currently lacking. It is imperative to discover safe and efficient treatment strategies to enhance the survival rate of RGCs and safeguard the visual function of individuals with glaucoma.

**FIGURE 1:**
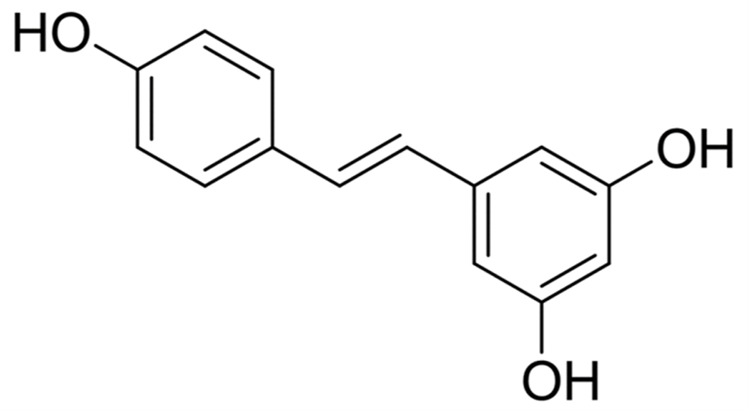
The chemical structure of resveratrol.

Resveratrol (C14H12O3, Figure 1), a natural polyphenol abundant in diverse plants like grapes, possesses antioxidant, anti-inflammatory, and neuroprotective characteristics. It has found extensive application in various eye-related disorders (Lancon et al., 2016; Bryl et al., 2022; Delmas et al., 2021). One potential mechanism of resveratrol-mediated optic nerve protection entails the activation of silent information regulator factor 1 (SIRT1) (Pallas et al., 2009), a protein extensively distributed in the nucleus and cytoplasm of the retina. SIRT1 plays a pivotal role in modulating the activity of various transcription factors and co-factors, influencing the expression of downstream genes, and regulating associated physiological and pathological processes (Zhou et al., 2018). Studies have demonstrated that the upregulation of SIRT1 exerts a protective effect against various eye diseases (Zhou et al., 2018; Shindler et al., 2007), and resveratrol can offer retinal neuroprotection in acute glaucoma models through the activation of SIRT1 (Chen et al., 2013; Luo et al., 2020; Luna et al., 2009). While certain preclinical studies have indicated favorable outcomes of resveratrol in ameliorating retinal ischemic injury in animal models of glaucoma, there remains a shortage of high-quality evidence validating resveratrol’s ability to confer neuroprotective effects in such models. Further exploration is warranted to elucidate the protective mechanism of resveratrol on the optic nerve in glaucoma.

Preclinical animal experiments serve as a vital connection in scientific research, bridging findings from the molecular and cellular levels to clinical trial investigations. Regrettably, discrepancies in animal modeling and other facets of preclinical studies have hindered the optimistic clinical translation of their results. Previous preclinical inquiries into the neuroprotective impacts of resveratrol in glaucoma models have exhibited notable variations in reported outcomes stemming from differences in modeling techniques and assessment criteria. This study aims to gather pertinent animal research, conduct meta-analyses of animal experiments, systematically assess the protective efficacy of resveratrol in glaucoma animal models, and appraise the potential clinical translational applications of resveratrol.

## 2 Methods

This study adhered to the Preferred Reporting Items for Systematic Review and Meta-Analysis (PRISMA) guidelines and was registered with PROSPERO (CRD42024535673).

### 2.1 Search strategy

We conducted a search of electronic databases from their inception up to 30 April 2024, which included Web of Science, Embase, PubMed, China Biomedical Database (CBM), China National Knowledge Infrastructure (CNKI), Wanfang Database (WF), and China Science Journal Database (VIP). Additionally, we will explore potential eligible articles from the reference lists of retrieved papers to mitigate any potential gaps in the research literature. Search keywords comprised a combination of MeSH terms and free-text words (Supplementary Table 1).

### 2.2 Eligibility criteria

According to the PICO principle, the studies included must meet the following criteria: (1) Population: Animal models of glaucoma-induced retinal injury, without restrictions on animal species, gender, age, or weight. (2) Intervention: Administration of resveratrol treatment, with no limitations on dosage, duration, or frequency. (3) Comparison: The control group should receive an equivalent carrier, physiological saline, or no treatment. (4) Outcome: Evaluation of the protective effects of resveratrol on the retina using outcome measures such as RGC survival rate, RGC apoptosis rate, visual function, retinal thickness, and retinal damage.

Exclusion criteria: (1) Clinical, *in vitro*, and computer simulation studies. (2) Studies where the control or treatment groups received a combination of resveratrol and other treatments. (3) Duplicate publications; in case of data duplication, the latest data will be retained. (4) Animal studies where experimental data cannot be retrieved.

### 2.3 Data extraction

The literature search was independently conducted by two researchers. Initially, irrelevant literature was excluded based on titles and abstracts. Subsequently, studies meeting the inclusion criteria were selected by reviewing the full text, and the following information was extracted: (1) Publication details including author(s) and publication year; (2) Details regarding animal species, gender, age, weight, and sample size; (3) Methods employed for establishing glaucoma models and administering anesthesia; (4) Information on the timing, dosage, route of administration, and control method of resveratrol; (5) Outcome measures. In cases where results are graphically presented, attempts were made to contact the corresponding authors of the research to obtain raw data. If unsuccessful, the data were processed using WebPlotDigitizer 4.5 (https://automeris.io/) to extract quantitative data from the graphs. When a study encompasses various doses and time points of administration, yielding multiple datasets for outcome indicators, the selection process for meta-analysis typically involves choosing the most efficacious dose or time point data. However, during dose-response analysis, data extraction entails utilizing information from distinct dose groups for comprehensive analysis. Any discrepancies in the data extraction procedure should be reconciled through consultation with a third researcher.

### 2.4 Quality assessment

Two assessors independently assessed and graded the included studies using the SYRCLE tool for evaluating bias in animal studies (Hooijmans et al., 2014). The types of biases considered encompass selection bias, performance bias, detection bias, attrition bias, reporting bias, and other potential biases. Assessment outcomes were categorized as “yes” for low risk of bias, “no” for high risk of bias, and “uncertain” for unclear risk of bias.

### 2.5 Statistical analysis

Statistical analysis was performed using STATA software version 17.0. The outcome measures, represented as continuous variables, were compared by reporting the standard mean deviation (SMD) and 95% confidence interval (CI) for overall effect sizes. A significance level of p < 0.05 denoted statistical significance. Heterogeneity within the study was assessed using I2 values. A fixed-effects model was employed when I2 was 50% or less, while I2 values exceeding 50% indicated significant heterogeneity, warranting sensitivity, and subgroup analyses to investigate potential sources of heterogeneity. In cases where significant heterogeneity persisted unresolved, a random-effects model was utilized. Publication bias was evaluated using Egger’s linear regression analysis and Begg’s rank correlation analysis. If publication bias was detected, trimming and filling methods were employed to address it.

## 3 Results

### 3.1 Study selection

**FIGURE 2:**
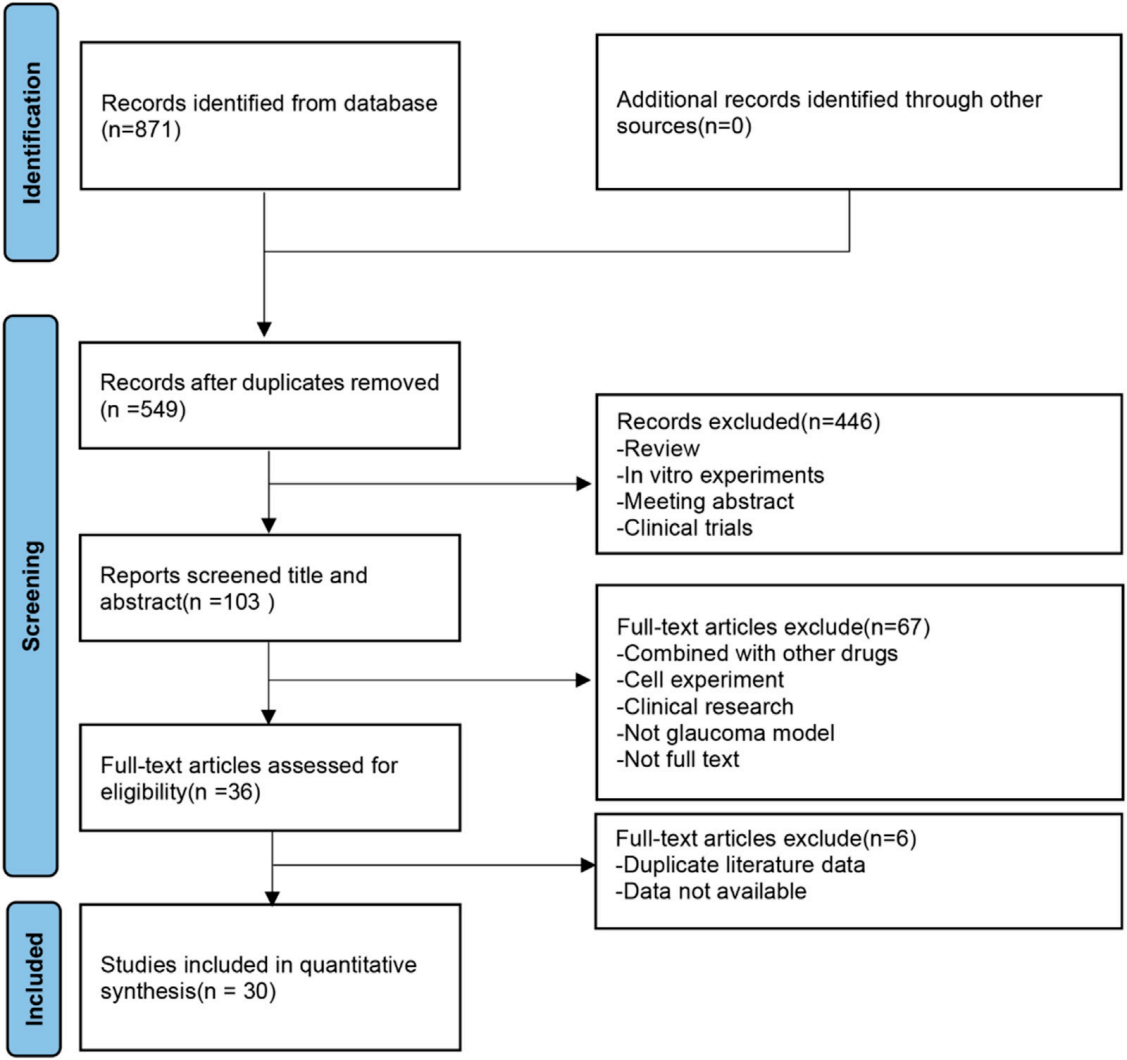
Flow diagram of the study selection process.

A total of 871 potentially relevant articles were retrieved from seven electronic databases: PubMed (112), Embase (378), Web of Science (167), CNKI (36), CBM (78), WF (43), and VIP (57). After removing duplicates, 549 articles remained. Subsequently, 446 irrelevant articles were excluded based on title and abstract reviews. Upon full-text assessment, 73 more articles were excluded, resulting in the inclusion of 30 articles. The research selection process is illustrated in Figure 2.

### 3.2 Characteristics of included studies

Out of 30 studies, 21 studies visually depicted their findings graphically, with 9 of these studies acquiring raw data directly from the corresponding author or first author. Furthermore, in 12 studies, two researchers used WebLotDigitizer 4.5 to extract data and took the average for meta-analysis. The 30 included studies involved a total of 559 animals, with 280 in the treatment group and 279 in the control group. Among these, 14 studies utilized 286 Sprague Dawley rats, 12 studies involved 211 C57BL/6J mice, 2 studies utilized 42 Wistar rats, 1 study employed 10 brown rats, and another study used 10 Agouti rats. Male animals were used in 24 studies, while 2 studies involved both male and female animals, and 4 studies did not specify the gender. The age of the animals was provided in 25 studies, and the weight was specified in 16 studies. Regarding the administration of resveratrol, 7 studies employed intravitreal injection, 17 studies utilized intraperitoneal injection, 4 studies employed oral or gavage administration, 1 study used eye drops, and 1 study did not specify the injection method. Concerning outcome measures, 23 studies reported on the survival or death of RGCs, 8 studies have reported the transcription factor Brn3 protein (Brn3a) that can be used to label and quantify RGCs (Nadal-Nicolas et al., 2009), 13 studies described changes in retinal thickness, 14 studies reported on apoptosis-related indicators of retinal tissue, 6 studies reported on A and B waves of electroretinography (ERG), 10 studies reported on inflammatory cytokine levels in local retinal tissue, and 11 studies reported on SIRT1 levels. Detailed characteristics of the included studies are presented in Table 1.

**TABLE 1:** Basic characteristics of the included studies.

Study (year)	Species (sex, age,n = treatment/model group, weight)	Modeling method	Resveratrol intervention (administration drug dose, duration)	Outcomes
Pirhan et al. (2016)	Wistar albino rats (male, adult, 12/14, NM)	Chronic elevation of IOP was induced in one eye of each animal by intracameral injection of hyaluronic acid.	Starting from glaucoma induction, 10 mg/kg resveratrol was administered via intraperitoneal injection daily for 6 weeks.	1. Survival status of RGC
				2. Death status of RGC
Seong et al. (2017)	C57BL/6J mice (male, 8 weeks, 11/11, 20–25 g)	Insert a 30 gauge needle into the anterior chamber of the right eye and add saline to increase the intraocular pressure to 60 mm Hg; Maintain HIOP for 60 min.	Mice were intraperitoneally injected with resveratrol (20 mg/kg) once per day for 5 days (2 days before modeling and until sacrifice).	1. Survival status of RGC
				2. Retinal thickness
				3. Caspase-
Chronopoulos et al. (2023)	C57BL/6J mice (male, 5 to 6-month-old, 8/8, NM)	Introduce the tip of a borosilicate glass micropipette (diameter 100 µ m) into the anterior chamber, connect it to a reservoir filled with Ringer’s solution through a silicon tube, and increase intraocular pressure (IOP) to 110 mm Hg for 45 min.	Mice were administered resveratrol via gavage (30 mg/kg) once a day for 9 days (1 day before modeling until 7 days after modeling).	1. Survival status of RGC
				2. Brn3a
Luo et al. (2018)	Sprague Dawley rats (male, 2–3 months, 14/14, NM)	The anterior chambers of both eyes were cannulated briefly, and the IOP in one eye was elevated above systolic blood pressure (approximately 110 mm Hg) for 60 min.	Rats were injected intraperitoneally with 250 mg/kg RES three times, respectively, from 1 day before modeling until 1 day after modeling.	1. Survival status of RGC
				2. Retinal thickness
				3. Bax, Bcl-2
				4.iNOS, COX2
				5. Brn3a
Pang et al. (2020)	Sprague Dawley rats (male, 2–3 months, 18/18, NM)	The anterior chambers of both eyes were cannulated briefly, and the IOP in one eye was elevated above systolic blood pressure (approximately 110 mm Hg) for 60 min	Rats were injected intraperitoneally with 25 mg/kg RES three times, respectively, from 1 day before modeling until 1 day after modeling.	1. Survival status of RGC
				2. Retinal thickness
				3. SIRT1
Cao et al. (2020)	C57BL/6J mice (male, adult, 15/15, NM)	Mice received 3 μL of 10-μm-diameter polystyrene microbeads via a 35-gauge needle, into the anterior chamber of the right eye.	Mice were administered 1 μL of 30-μM RSV by injection into the right eye with a 35-gauge needle in the vitreous body on the same day after the microbead injection.	1. Death status of RGC
				2. SIRT1
				3. Brn3a
Xie et al. (2023)	C57BL/6 mice (male,6–8-week, 6/3, NM)	A 30-gauge needle was inserted into the anterior chamber of the right eye with a balanced salt solution. The IOP of the right eye was maintained above the systolic pressure (∼110 mm Hg) for 60 min.	Mice were intraperitoneally injected with RES (25 mg/kg) for five consecutive days before modeling and then intraperitoneally injected again immediately after modeling.	1. Survival status of RGC
				2. Retinal thickness
				3. Caspase-1
				4. IL-1β
				5. Brn3a
Vin et al. (2013)	Sprague Dawley rats (male, adult, 6/6, 200 g)	The anterior chamber of the right eye of each rat was cannulated with a 30-gauge sterile needle connected to an elevated isotonic sterile saline bag. The intraocular pressure was raised to 70e80 mm Hg for 45 min.	Rats were intraperitoneally injected with resveratrol (30 mg/kg) once per day for 5 days (2 days before modeling injury until 2 days after modeling).	1. Retinal thickness
				2. A-wave and b-wave of the ERG
Feng et al. (2024)	C57BL/6 mice (male, 6–8-week, 14/10, NM)	Cannulation with a 32-gauge needle of the anterior chamber was executed to raise the IOP to approximately 110 mmHg, which was maintained for 60 min.	Mice were intraperitoneally injected with resveratrol (20 mg/kg) once per day from 2 days before modeling until sacrifice.	1. Survival status of RGC
				2. Caspase-1
				3. IL-1β
				4. A-wave and b-wave of the ERG
Wu et al. (2020)	Sprague Dawley rats (male, 2–3 months, 6/6, 250 g)	The IOP of the left eye was increased to 110 mmHg for 60 min by placing a needle in the anterior chamber and elevating a saline reservoir containing 0.9% NaCl.	Resveratrol then was applied by intraperitoneal injections at 1 day before, at the time of, and 1 day after modeling.	1. RGC survival rate
				2. SIRT1
				3. Brn3a
Xia et al. (2020)	Sprague Dawley rats (male, 8 weeks, 24/24, 232 ± 28 g)	A needle connected to a saline bag about 150 cm high (110 mmHg pressure) was inserted into the anterior chamber of the rat eyes.	Rats in the resveratrol group received intraperitoneal injection of resveratrol (250 mg/kg) at the same time point 24 h before modeling, and thereafter at 15 min and at 48 h.	1. Survival status of RGC
Deng et al. (2020)	Sprague Dawley rats (male, 2 months, 4/4, NM)	The anterior chambers of both eyes were briefly cannulated, and the IOP in one eye was elevated above systolic blood pressure (approximately 110 mmHg) for 60 min.	Rats were injected with RES three times (250 mg/kg, intraperitoneal injection.), 1 day before, at the time of, and 1 day after modeling.	1. Retinal thickness
				2. iNOS, COX2
Zhang et al. (2018)	Sprague Dawley rats (male, 4–6-week, 6/6, 100–150 g)	The micro-magnetic beads (15–25 μL, diameter ≈ 9 μm) were slowly injected into the anterior chamber from the peripheral area of the cornea with a 30-gauge needle.	A dose of 20 mg/kg/d was given with intragastric administration from day 1 of post-operation and maintained daily for 4 weeks.	1. Survival status of RGC
				2. Death status of RGC
				3. Retinal thickness
Ji et al. (2024)	C57BL/6 mice (NM, 6–8-week, 12/12, NM)	The anterior chamber of the right eye was cannulated with a 30-gauge needle attached to a normal saline reservoir which was elevated to maintain an intraocular pressure (IOP) above systolic pressure (approximately 110 mmHg) for 1 h	Mice were intraperitoneally injected with RES (20 mg/kg) once per day for 5 days (2 days before modeling and until sacrifice).	1. Survival status of RGC
				2. Bax, Bcl-2
				3. Caspase-3
				4. A-wave and b-wave of the ERG
				5. IL-6
				6. SIRT1
				7. Brn3a
Prasetya et al. (2023)	*Rattus Norvegicus* (NM, 6–8-week, 5/5, 250–300 g)	The IOP was increased by injecting a balanced salt solution (BSS) into the anterior chamber using a cannula of 30 G. The IOP was maintained at 110 mmHg for 60 min.	Rattus was injected with Resveratrol 100 µM in 2 µL intravitreal after modeling.	1. Bax
				2. Caspase-3
Zhao et al. (2022)	C57BL/6 mice (NM, NM, 12/12, NM)	A 32G needle was used to puncture at 1 mm from the lateral corner iris edge, followed by the injection of 20 nmol N-methyl-d-aspartic acid solution into the vitreous cavity using a 10 µL microsyringe.	After modeling, mice were injected with 20 mg/kg resveratrol solution, and 24 h later, 20 mg/kg resveratrol solution was repeatedly injected.	1. Survival status of RGC
				2. Death status of RGC
				3. Retinal thickness
				4. SIRT1
Seong et al. (2022)	C57BL/6 mice (male, 8-week, 3/4, 20–25 g)	The anterior chamber was cannulated with a 30-gauge needle to increase the IOP to 60 mm Hg for 60 min.	Mice were intraperitoneally injected with RES (20 mg/kg) once per day for 5 days (2 days before modeling and until sacrifice).	1. Death status of RGC
Chang et al. (2018)	Sprague Dawley rats (male, NM, 6/6, 200 ± 20 g)	The anterior chambers were briefly cannulated with a 20-gauge indwelling needle, and the IOP was elevated above systolic blood pressure (approximately 110 mmHg) for 60 min.	Rattus were injected with Resveratrol (0.5 nmol/L) 5 µL in intravitreal 0.5 h before modeling.	1. Death status of RGC
				2. Retinal thickness
				3. Caspase-3
				4. Bcl-2
Liu et al. (2013)	Wistar rats (male, 6–8-week, 8/8, NM)	The anterior chambers were briefly cannulated with a 30-gauge indwelling needle, and the IOP was elevated above systolic blood pressure (approximately 120 mmHg) for 60 min.	Rats were injected with Resveratrol (0.5 nmol) in intravitreal 15 min before modeling.	1. Survival status of RGC
				2. B-wave of the ERG
				3. iNOS
Chen (2018)	Sprague Dawley rats (male,2–3 months, 4/4, 250–320 g)	The anterior chambers were briefly cannulated, and the IOP was elevated above systolic blood pressure (approximately 110 mmHg) for 60 min.	Rats were injected with RES three times, 1 day before (250 mg/kg, intraperitoneal injection.), at the time of (250 mg/kg, intraperitoneal injection.), and 1 day after modeling (300 mg/kg, intraperitoneal injection).	1. Retinal thickness
				2. iNOS, COX2
Luo et al. (2020)	C57BL/6 mice (male, 8–10-week, 9/9, 20–24 g)	The anterior chambers were briefly cannulated with a 30-gauge indwelling needle and maintained high intraocular pressure for 60 min.	Mice were injected with Resveratrol (100 μM) in intravitreal 1 day before modeling.	1. Survival status of RGC
				2. B-wave of the ERG
				3. Bax, Bcl-2
				4. SIRT1
Xiong (2021)	Sprague Dawley rats (male, 8–10 weeks, 10/10, 180–200 g)	The 532 nm laser is used to burn the superficial venous ring and branches around the sclera edge, with approximately 100 points burned in each eye. After 7 days of modeling, the intraocular pressure is measured, and if the intraocular pressure is greater than 22 mmHg, the modeling is successful	After successful modeling, rats were intraperitoneally injected with RES (20 mg/kg) once a day for 21 consecutive days	1. Bax, Bcl-2
				2. IL-1β, IL-6
				3. SIRT1
				4. Caspase-3
Li (2012)	C57BL/6 mice (male, NM, 5/7, NM)	The anterior chambers were briefly cannulated with a 33-gauge indwelling needle, and the IOP was elevated above systolic blood pressure (approximately 150 mmHg) for 60 min.	Mice were fed RES (20 mg/kg) once a day from 2 days before modeling until sacrifice.	1. Survival status of RGC
				2. Retinal thickness
				3. Brn3a
Ji (2016)	Sprague Dawley rats (male, 2–3 months, 5/5, 250–300 g)	The anterior chambers were briefly cannulated with a 30-gauge indwelling needle and maintained high intraocular pressure for 60 min.	Mice were injected with Resveratrol (4 ul 10 μM) in the intravitreal after modeling.	1. SIRT1
Ji (2022)	C57BL/6 mice (male, 6–8-week, 5/7, 20–22 g)	The anterior chambers were briefly cannulated with a 32-gauge indwelling needle and maintained high intraocular pressure for 60 min.	Mice were intraperitoneally injected with RES (20 mg/kg) once per day for 5 days (2 days before modeling and 2 days after modeling).	1. Survival status of RGC
				2. Retinal thickness
				3. Bax, Bcl-2
				4. Caspase-3
				5. IL-6
				6. A-wave and b-wave of the ERG
				7. SIRT1
				8. Brn3a
Zhou et al. (2016)	Sprague Dawley rats (male, 2–3 months, 10/10, 250–320 g)	The anterior chambers were cannulated and maintained high intraocular pressure for 60 min.	Rats were injected with RES three times (20 mg/kg), 1 day before, 15 min after, and 1 day after modeling.	1. SIRT1
Guowu et al. (2020)	Sprague Dawley rats (male and female, 6–8 weeks, 20/20, 180–210 g)	Inject fresh mixed HCCS (7 μL) into the anterior chamber using a 31-gauge needle to maintain intraocular pressure at 22 mm Hg.	After successful modeling, rats were given resveratrol (80 mg/kg) orally for 12 weeks	1. Death status of RGC
				2. Bax, Bcl-2
				3. Caspase-3
He et al. (2021)	Sprague Dawley rats (male and female, 8 weeks, 10/10, 180–220 g)	The surgical cauterizer gently cauterizes the scleral vein, with the proximal corneal end blood vessels expanding and filling, and the distal corneal end blood vessels disappearing. After 7 days of surgery, the intraocular pressure is greater than 21 mmHg or 5 mmHg higher than that of the non-surgical eye.	After successful modeling, resveratrol was injected intraperitoneally once a day (40 mg/kg) for 8 consecutive days.	1. Survival status of RGC
Shamsher et al. (2022)	Dark Agouti rats (NM, NM, 5/5, NM)	The ocular hypertension rat model was induced with the injection of 1.85 M normal saline solution in two episcleral veins of the left eye of rats.	Resveratrol nanoparticles were given topically as an eye drop daily for 3 weeks from induction	1. Survival status of RGC
				2. Death status of RGC
Zhu et al. (2018)	C57BL/6 mice (male, NM, 7/6, NM)	The model was established by increasing IOP to 95 mmHg for 90 min by inserting a needle, which was connected to the elevated balanced-salt solution, into the anterior chamber of the right eye of mice.	The treatment was done by intraperitoneal injection and was initiated on the day of IOP elevation, and repeated daily for 1 week and 4 weeks respectively.	1. Death status of RGC

IOP: intraocular pressure; RGC: retinal ganglion cells; ERG: electroretinography; RSV: resveratrol.

### 3.3 Study quality

**FIGURE 3:**
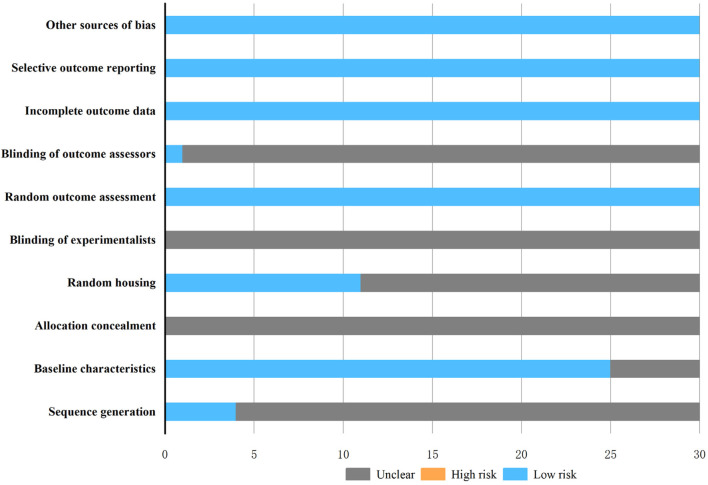
Risk of bias graph.

According to Figure 3, the quality assessment of the 30 studies was conducted using SYRCLE’s risk of bias tool, with 2 studies scoring 4 points (Xiong, 2021; Xia et al., 2020), 19 studies scored 5 points (Deng et al., 2020; Chronopoulos et al., 2023; Luo et al., 2018; Pang et al., 2020; Cao et al., 2020; Vin et al., 2013; Feng et al., 2024; Wu et al., 2020; Zhang et al., 2018; Ji et al., 2024; Chang et al., 2018; Liu, 2013; Chen, 2018; Li, 2012; Ji, 2022; Ji, 2016; He et al., 2021; Shamsher et al., 2022; Zhu et al., 2018),7 studies scored 6 points (Seong et al., 2017; Xie et al., 2023; Prasetya et al., 2023; Zhao et al., 2022; Seong et al., 2022; Luo, 2020; Academic Exchange Conference, 2016),2 studies scored 7 points (Pirhan et al., 2016; Guowu et al., 2020). Among the 30 included studies, only 4 studies mentioned random grouping, 25 studies detailed baseline characteristics, 11 studies outlined animal random housing, all random outcome assessments of the studies were rated as low-risk, but only 1 study documented the blinding of outcome assessors, and no study elucidated how blinding was implemented in allocation concealment and experimentalists. All studies were deemed low-risk in terms of incomplete outcome data, selective outcome reporting, and other sources of bias. The comprehensive evaluation results are presented in Supplementary Table 2.

### 3.4 Effectiveness

#### 3.4.1 Primary outcomes

##### 3.4.1.1 Survival of RGCs

**FIGURE 4:**
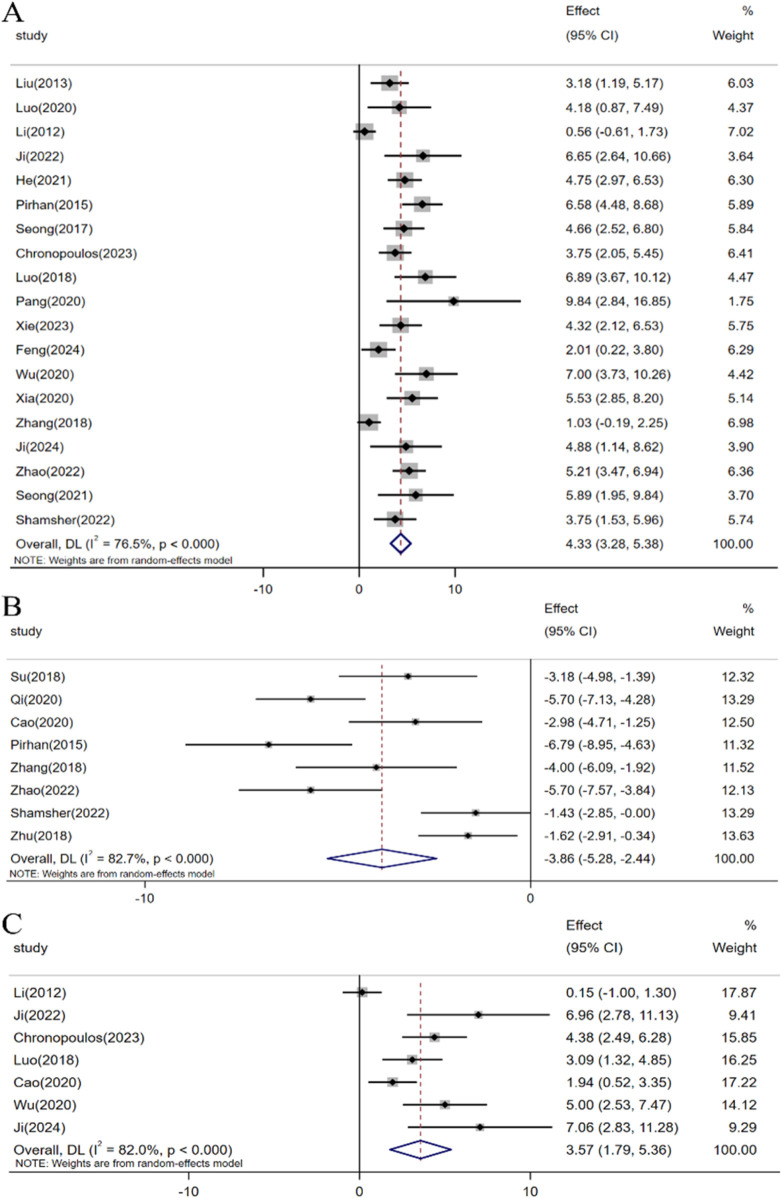
Forest plot: effect of resveratrol on **(A)** Survival status of RGC, **(B)** Death status of RGC, **(C)** Brn3a.

In the included studies, a total of 23 investigations assessed the impact of resveratrol on RGCs in glaucoma models. Meta-analyses involving 19 studies indicated that resveratrol intervention significantly enhanced the survival rate of RGCs under elevated intraocular pressure [n = 231, SMD: 4.33 (95% CI: 3.28, 5.38), *p* < 0.05; heterogeneity: I2 = 76.5%, *p* < 0.05, Figure 4A]. Moreover, meta-analyses of 8 studies demonstrated that resveratrol could decrease the number of RGC deaths [n = 147, SMD: −3.86 (95% CI: −5.28, −2.44), *p* < 0.05; heterogeneity: I2 = 82.7%, *p* < 0.05, Figure 4B]. A meta-analysis of 7 studies revealed that resveratrol intervention led to an increase in Brn3a-labeled RGCs compared to the control group [n = 80, SMD: 3.57 (95% CI: 1.79, 5.36), *p* < 0.05; heterogeneity: I2 = 82.0%, *p* < 0.05, Figure 4C].

##### 3.4.1.2 Retinal thickness

**FIGURE 5:**
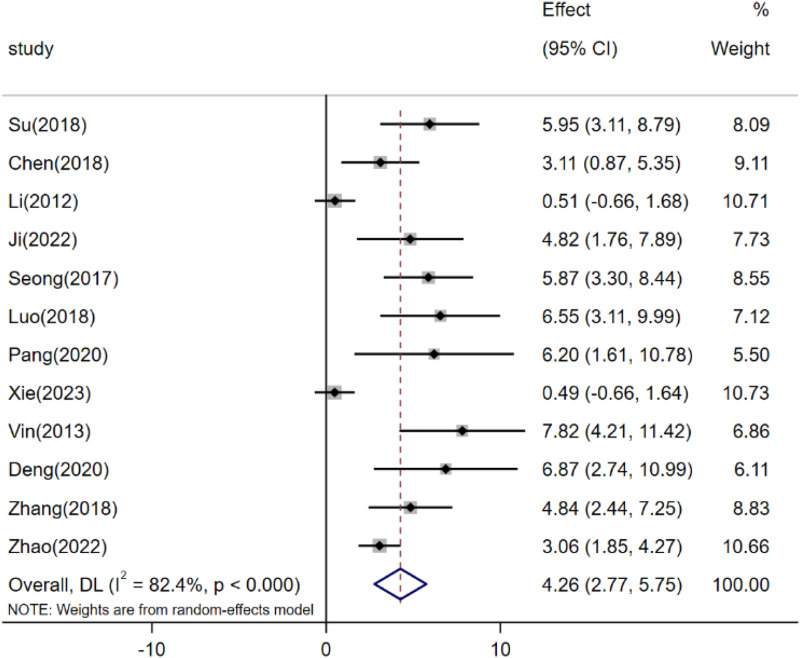
Forest plot: effect of resveratrol on Retinal thickness.

A meta-analysis of 12 studies revealed that resveratrol, when compared to the control group, can ameliorate retinal thickness thinning in high intraocular pressure conditions [n = 138, SMD: 4.26 (95% CI: 2.77, 5.75), *p* < 0.05; heterogeneity: I2 = 82.4%, *p* < 0.05, Figure 5].

#### 3.4.2 Secondary outcomes

##### 3.4.2.1 Retinal function

**FIGURE 6:**
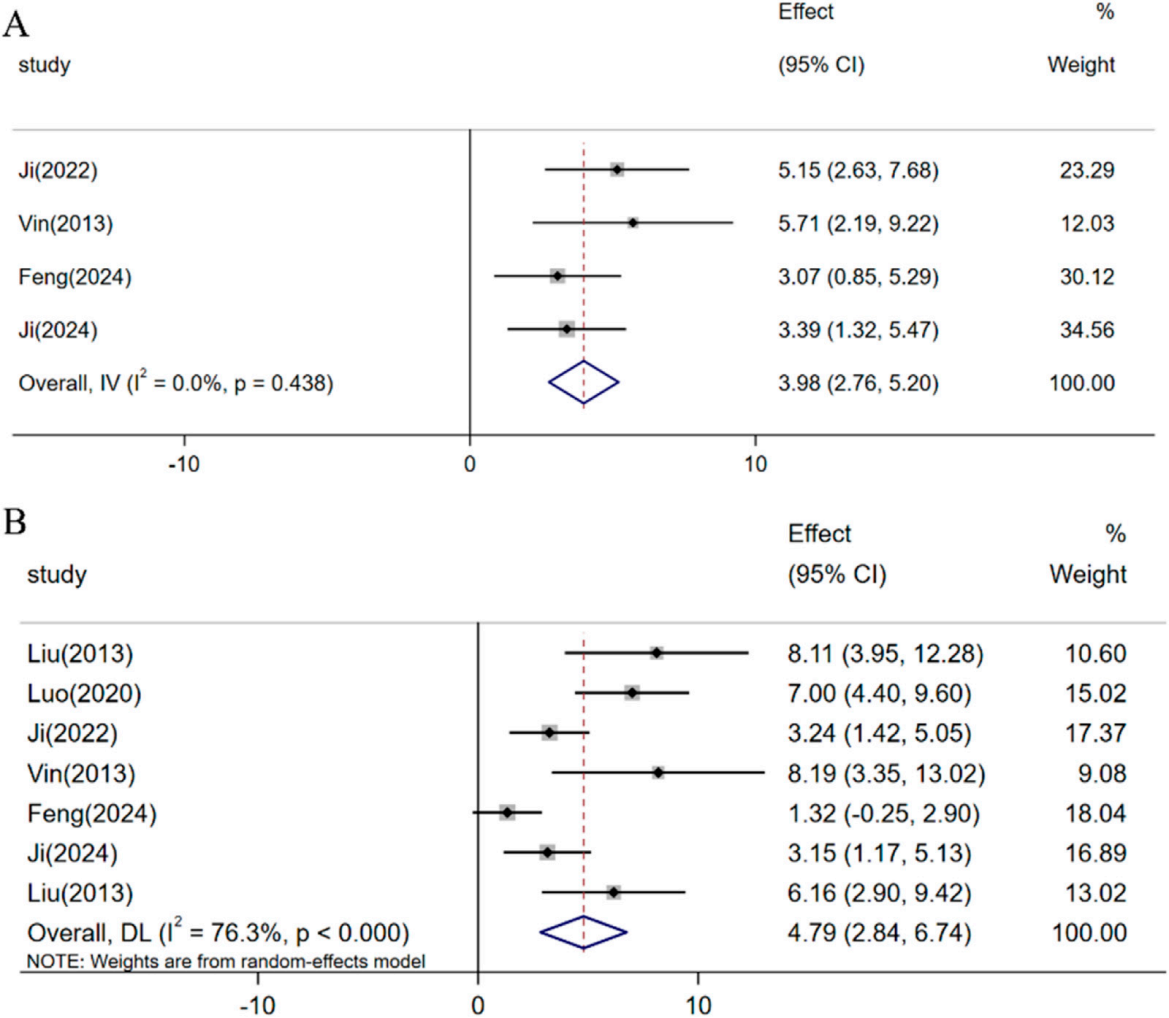
Forest plot: effect of resveratrol on **(A)** A-wave of the ERG and **(B)** B-wave of the ERG.

Based on the dark adaptation flash ERG data included in the study, a meta-analysis of 4 studies indicated that resveratrol can increase the A-wave amplitude [n = 38, SMD: 3.98 (95% CI: 2.76, 5.20), *p* < 0.05; heterogeneity: I2 = 0.0%, *p* = 0.438, Figure 6A]. Similarly, a meta-analysis of 7 studies demonstrated that resveratrol can also elevate the B-wave amplitude [n = 76, SMD: 4.79 (95% CI: 2.84, 6.74), *p* < 0.05; heterogeneity: I2 = 76.3%, *p* < 0.05, Figure 6B]. Consequently, compared with the control group, resveratrol can increase the amplitude of “a” and “b” waves in ERG.

##### 3.4.2.2 The expression level of SIRT1 protein in the retina

**FIGURE 7:**
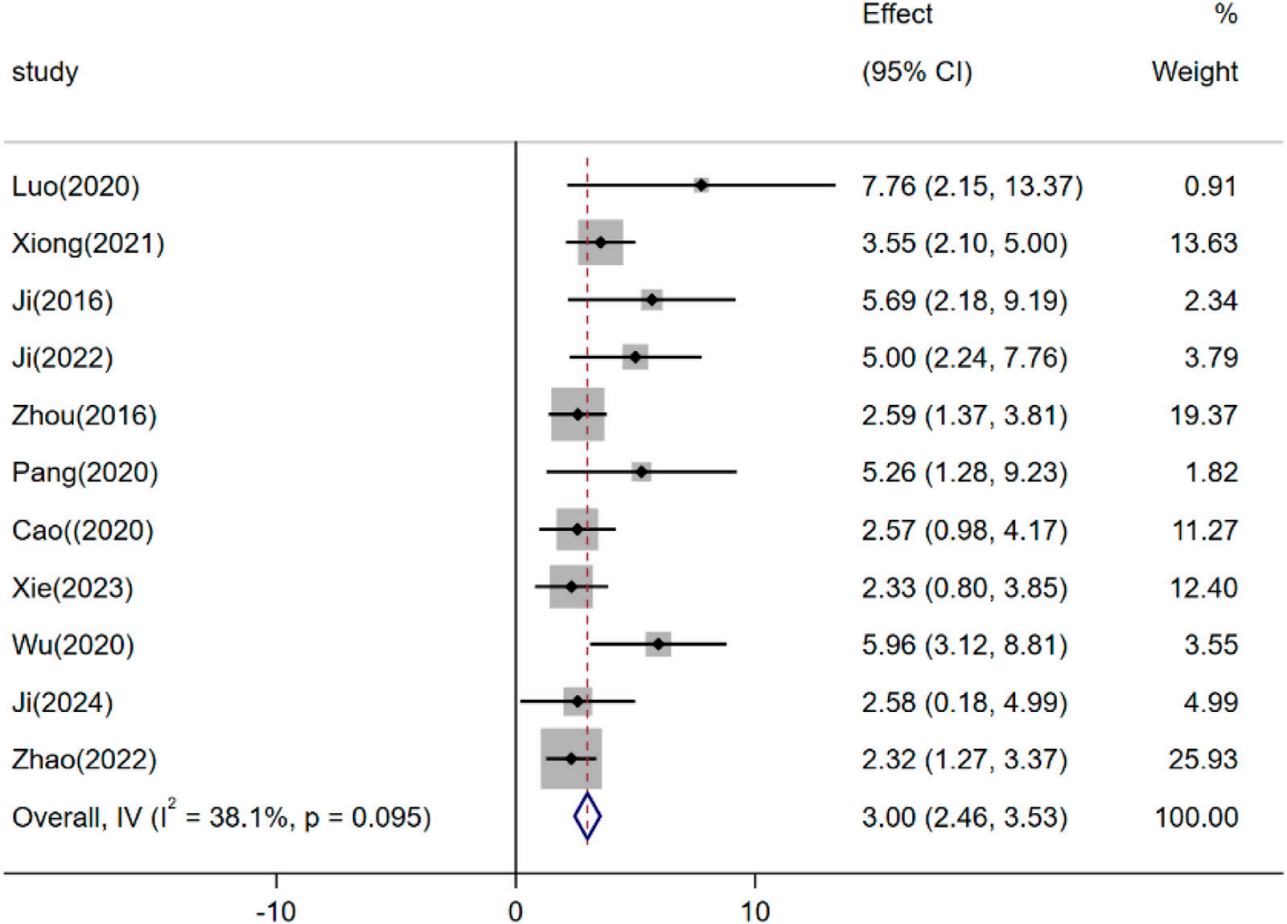
Forest plot: effect of resveratrol on SIRT1.

Incorporating data from 11 studies that assessed SIRT1 protein expression levels, the collective meta-analysis demonstrated that resveratrol can enhance the upregulation of SIRT1 protein expression [n = 136, SMD: 3.00 (95% CI: 2.46, 3.53), *p* < 0.05; heterogeneity: I2 = 38.1%, *p* = 0.095, Figure 7].

##### 3.4.2.3 Expression levels of inflammatory factors in retinal tissue

**FIGURE 8:**
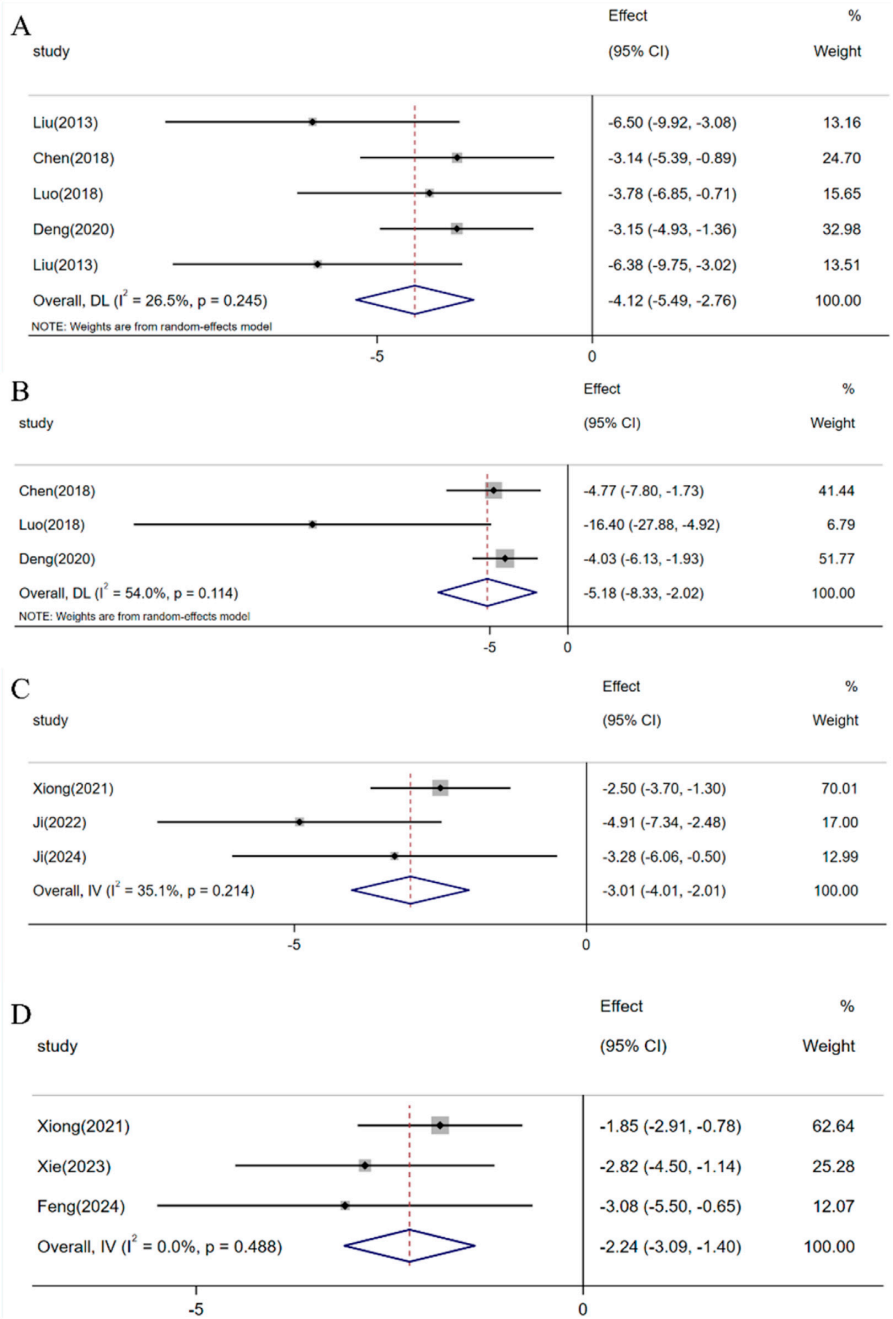
Forest plot: effect of resveratrol on **(A)** iNOS, **(B)** COX-2, **(C)** IL-6, **(D)** IL-1β.

A meta-analysis of 4 studies indicated that resveratrol can decrease the level of the inflammatory cytokine iNOS [n = 36, SMD: −3.65 (95% CI: −4.84, −2.46), *p* < 0.05; Heterogeneity: I2 = 5.8%, *p* = 0.364, Figure 8A]. Another meta-analysis involving 3 studies demonstrated that resveratrol can lower the level of the inflammatory factor COX-2 [n = 26, SMD: −5.18 (95% CI: −8.33, −2.02), *p* < 0.05; Heterogeneity: I2 = 54%, *p* = 0.114, Figure 8B]. Similarly, a meta-analysis of 3 studies revealed that resveratrol can decrease the level of the inflammatory factor IL-6 [n = 38, SMD: −3.01 (95% CI: −4.01, −2.01), *p* < 0.05; Heterogeneity: I2 = 34.1%, *p* = 0.214, Figure 8C]. Lastly, an analysis of 3 studies found that resveratrol can reduce the level of the inflammatory factor IL-1β [n = 39, SMD: −2.24 (95% CI: −3.09, −1.40), *p* < 0.05; Heterogeneity: I2 = 0.0%, *p* = 0.488, Figure 8D].

##### 3.4.2.4 Expression levels of apoptosis-related proteins in retinal tissue

**FIGURE 9:**
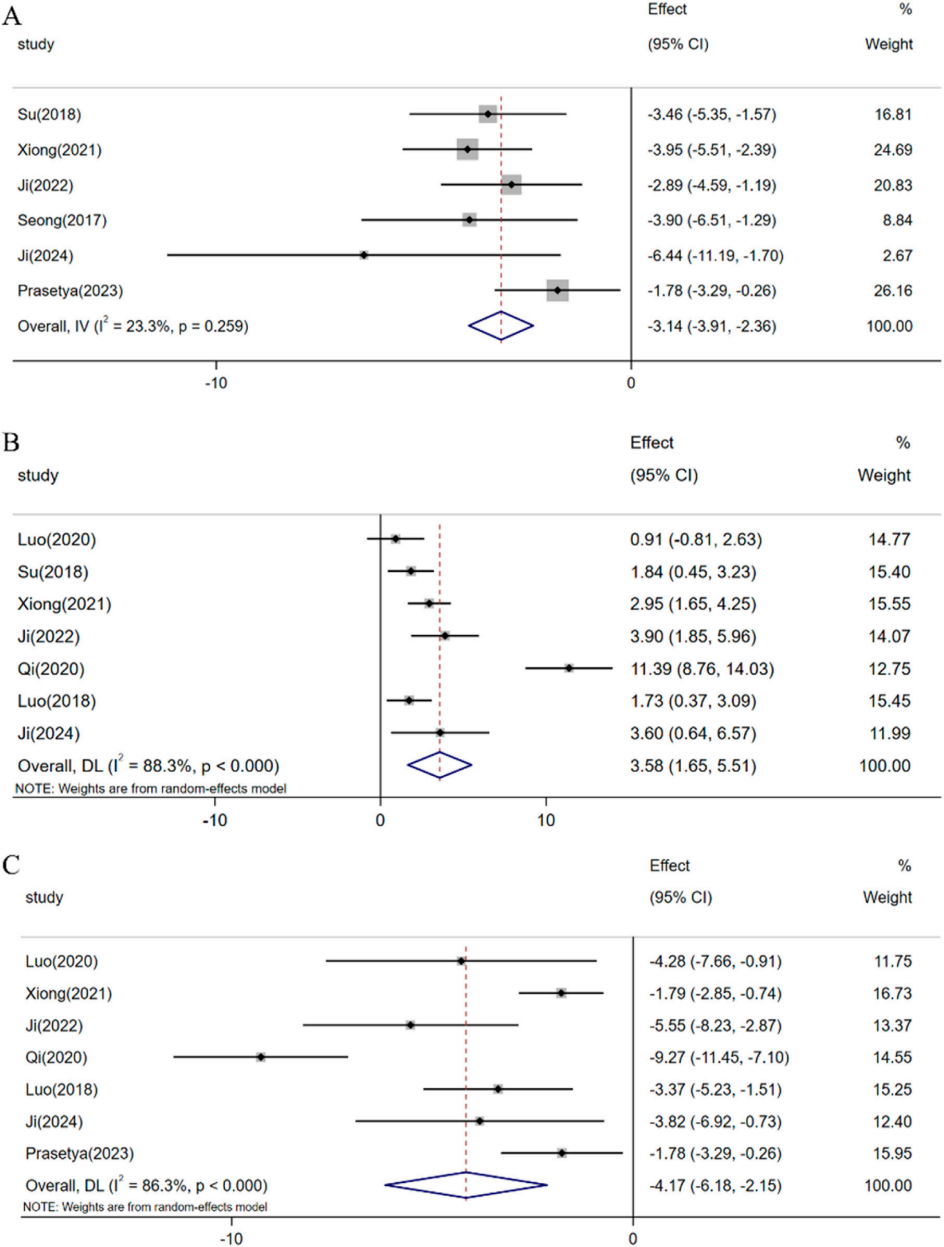
Forest plot: effect of resveratrol on **(A)** Capase-3, **(B)** Bcl-2, **(C)** Bax.

In the evaluation of 6 studies, the combined meta-analysis revealed that resveratrol can induce a reduction in the expression level of Caspase-3 protein in rat retinal tissue [n = 68, SMD: −3.14 (95% CI: −3.91, −2.36), *p* < 0.05; Heterogeneity: I2 = 23.3%, *p* = 0.259, Figure 9A]. Furthermore, meta-analysis results from 7 studies illustrated that resveratrol can facilitate an increase in Bcl-2 protein expression levels in retinal tissue [n = 108, SMD: 3.58 (95% CI: 1.65, 5.51), *p* < 0.05; heterogeneity: I2 = 88.3%, *p* < 0.05, Figure 9B]. Concurrently, the meta-analysis findings from 7 studies indicated that resveratrol can impede the upregulation of Bax protein expression in retinal tissue [n = 106, SMD: −4.17 (95% CI: −6.18, −2.15), *p* < 0.05; heterogeneity: I2 = 86.3%, *p* < 0.05, Figure 9C].

### 3.5 Sensitivity analysis

In our analysis of the main outcome measures with significant heterogeneity, we systematically assessed the impact of each study’s exclusion on the combined effects related to RGC survival, RGC death, Brn3a, and retinal thickness. Notably, after excluding data from Pirhan (2015) and Zhang (2018), the range of combined effect sizes for RGC survival spanned from 4.17 (95% CI: 3.12, 5.21) to 4.55 (95% CI: 3.51, 5.58). Similarly, upon excluding data from Pirhan (2015) and Shamsher (2022), the combined effect sizes for RGC mortality varied between −3.47 (95% CI: −4.87, −2.08) and −4.22 (95% CI: −5.68, −2.76). Following the exclusion of Ji (2024) and Li (2012), the combined effect sizes for Brn3a ranged from 3.19 (95% CI: 1.40, 4.98) to 4.09 (95% CI: 2.60, 5.78). Moreover, after excluding data from Vin (2013) and Li (2012), the combined effect sizes for retinal thickness varied from 3.97 (95% CI: 2.49, 5.44) to 4.70 (95% CI: 3.15, 6.25).

### 3.6 Subgroup analysis

Given the substantial heterogeneity observed between studies, we conducted subgroup analyses on RGC survival, RGC mortality, Brn3a, and retinal thickness, focusing on modeling methods, animal species, administration methods, and dosages. Our findings suggest that administration methods, dosages, and timing could potentially contribute to the observed heterogeneity in RGC survival. For RGC mortality, our analysis indicates that animal species, administration methods, and dosages may serve as possible sources of heterogeneity. Regarding Brn3a, the dosage and timing of administration are identified as potential sources of heterogeneity. Lastly, for retinal thickness, animal species and administration time are highlighted as potential sources of heterogeneity. The detailed results are presented in the attached Supplementary Table 3.

### 3.7 Publication bias

**FIGURE 10:**
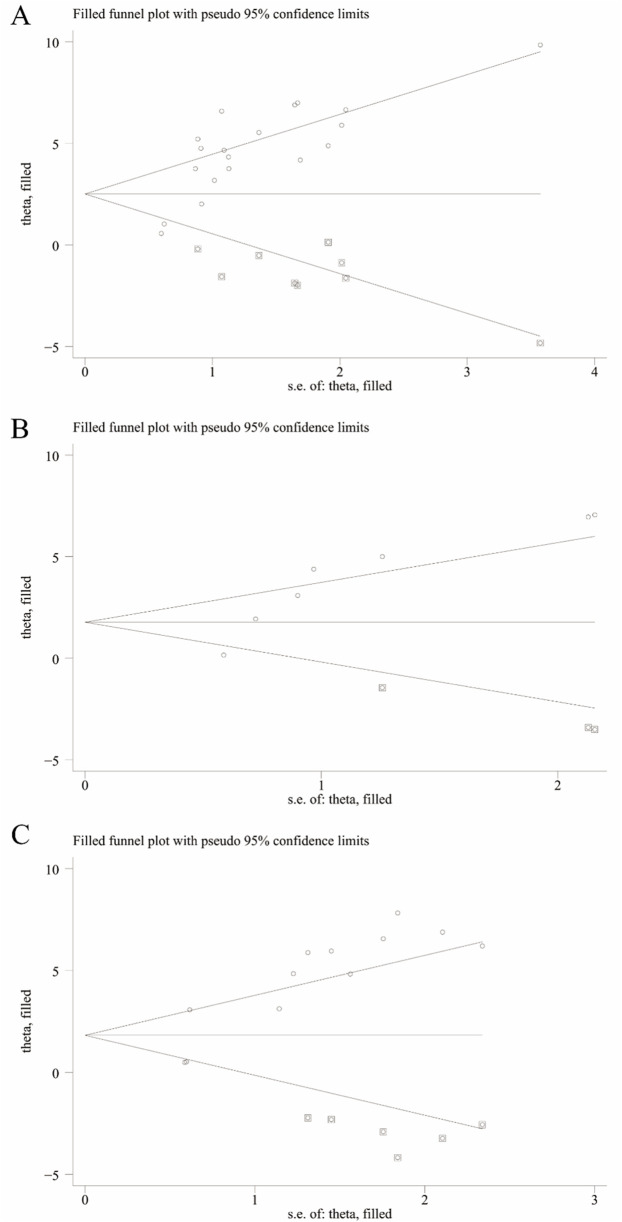
Trim-and-fill analysis for **(A)** Survival status of RGC, **(B)** Brn3a, and **(C)** Retinal thickness.

We utilized Egger’s test and Begg’s test to assess the potential publication bias in studies concerning RGC survival, RGC mortality, Brn3a, and retinal thickness. The findings revealed statistically significant publication bias for RGC survival, Brn3a, and retinal thickness, while no statistically significant publication bias was detected for RGC mortality (Supplementary Figure 1). Furthermore, employing pruning and filling methods, we conducted statistical analyses on studies that might have overlooked aspects related to RGC survival, Brn3a, and retinal thickness. The results suggest that the absence of certain research data does not significantly impact the overall consolidation effect, as depicted in Table 2 and Figure 10.

**TABLE 2:** The results from the trim-and-fill analysis.

	Before trim and fill			After trim and fill		
Parameter	P value	SMD	NO. studies	P value	SMD	NO. studies
Survival status of RGC	P < 0.05	4.33	19	P < 0.05	15.96	28
Brn3a	P < 0.05	3.57	7	P < 0.05	7.75	10
Retinal thickness	P < 0.05	4.26	12	P < 0.05	8.57	18

### 3.8 Dose effect analysis of resveratrol dose and RGC survival quantity

**FIGURE 11:**
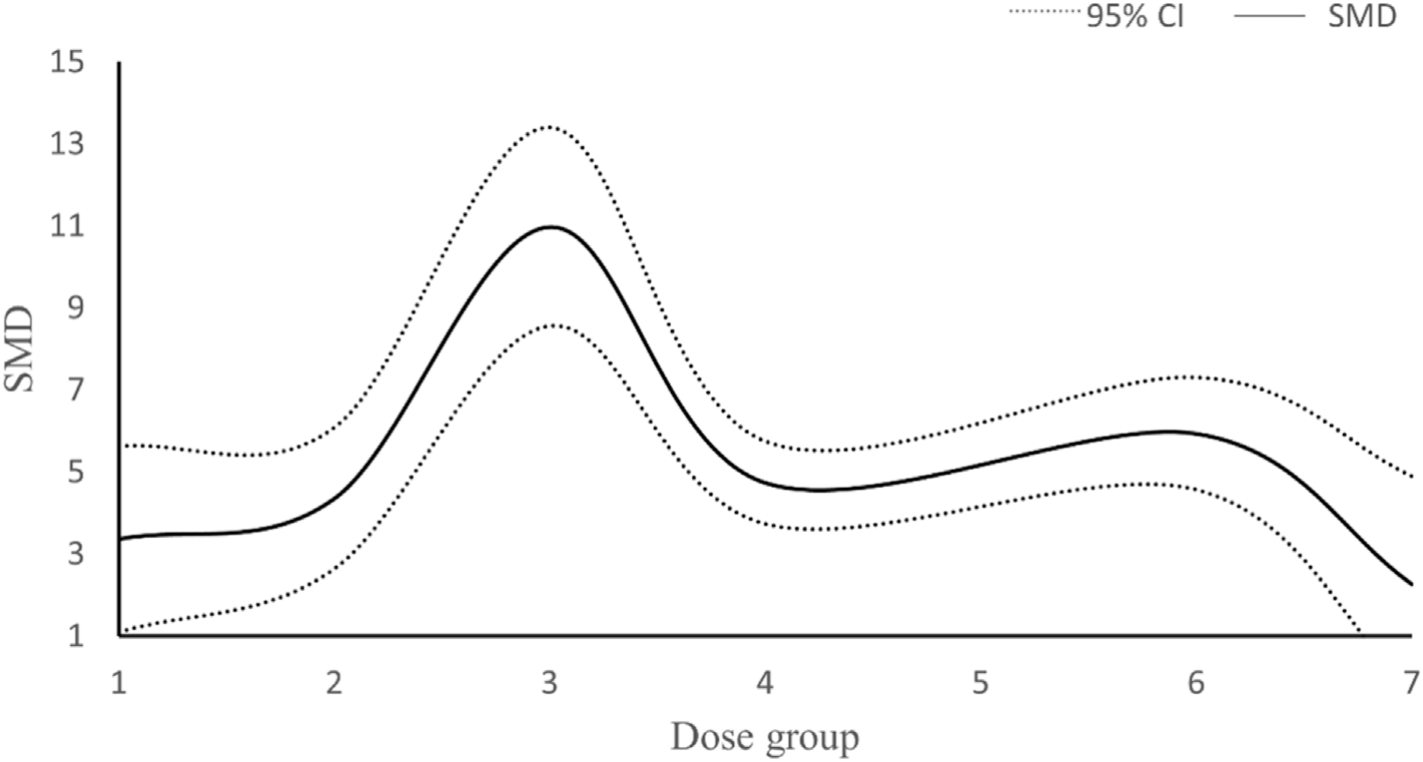
Dose-response curve of resveratrol in improving survival status of RGC. Dose group: (1) dose≤80 mg/kg, (2) 80<dose≤160 mg/kg, (3) 160<dose≤ 240 mg/kg, (4) 240<dose≤ 320 mg/kg, (5) 320<dose≤400 mg/kg, (6) 400<dose≤480 mg/kg, (7) >480dose mg/kg.

In our study, data from a total of 23 studies were analyzed concerning the dosage of resveratrol and its impact on RGC survival. Three studies were excluded from the analysis: one due to unclear dosing information and four due to the use of inconsistent dosage units. Among the remaining 18 studies, doses were administered in mg/kg, with 5 studies employing varying doses of resveratrol. Subsequently, the total dosage of resveratrol was categorized into 7 intervention methods. The research outcomes presented in Figure 11 suggest a non-linear correlation between resveratrol dosage and RGC survival. Notably, the maximum effect was observed when the total dose of resveratrol ranged between 160–240 mg/kg.

## 4 Discussion

### 4.1 Effectiveness

After synthesizing and evaluating data from 30 preclinical studies, our meta-analysis indicates that resveratrol exhibits promising therapeutic potential in the management of glaucoma-related retinal injuries. Notably, resveratrol demonstrates a protective effect by modulating key outcome measures in glaucoma retinal injury models. This includes enhancing the survival rate of RGCs, reducing the mortality rate of these cells, boosting Bra3a expression, and curbing retinal thinning. Furthermore, resveratrol shows efficacy in reversing retinal dysfunction induced by elevated intraocular pressure, as evidenced by increased A-wave and B-wave amplitudes. However, our meta-analysis uncovered significant heterogeneity in the primary indicators of RGC survival, RGC mortality, Bra3a expression, and retinal thickness. Despite conducting sensitivity analyses, the specific sources of this heterogeneity remained elusive. Subgroup analyses suggest that variations in animal species, administration methods, dosage, and timing of administration may contribute to this heterogeneity. To address potential publication biases in the assessment of RGC survival, Bra3a expression, and retinal thickness, we employed pruning and imputation techniques to estimate missing studies and data. The analysis indicates that publication bias does not significantly impact the robustness of our findings.

### 4.2 Potential mechanism

In glaucoma, optic nerve damage primarily manifests as structural alterations in the optic nerve head and progressive loss of RGC axons, resulting in distinct visual field deficits and impaired vision. A relatively recent discovery, the SIRT1 protein, is associated with ocular diseases, including those affecting the eyes (Wu et al., 2020). It plays a role in cellular stress response and cell survival (Porcu and Chiarugi, 2005). Upregulation of SIRT1 exhibits a protective effect against various ocular diseases (Zhou et al., 2018). Upon optic nerve damage, SIRT1 activation can enhance the survival of RGCs and mitigate inflammatory responses (Zuo et al., 2013; O'Neill et al., 2024). Its mechanism of action may involve the increased expression of the mitochondrial enzyme succinate dehydrogenase and the promotion of deacetylation of PGC-1α, a coenzyme crucial in mitochondrial function (Chen et al., 2013; O'Neill et al., 2024). Resveratrol, a natural polyphenolic compound known for boosting SIRT1 activity, can provide neuroprotection to RGCs in retinal IR injury, with this protective effect being attenuated by SIRT1 inhibitors (Luo et al., 2020). The meta-analysis comprising 11 studies revealed that resveratrol can enhance the expression of SIRT1 protein, thereby exerting a neuroprotective impact on the optic nerve.

The neuroprotective effects of resveratrol could also be associated with various other factors. Optic nerve damage in glaucoma involves multiple intertwined mechanisms such as inflammation and cellular apoptosis. Within the injured retina, pathogenic and reparative processes coexist during the inflammatory cascade. A controlled level of inflammatory response plays a pivotal role in preserving the retinal and neighboring environment’s homeostasis. However, an excessive inflammatory response can trigger a cascade of irreversible degenerative conditions including optic nerve damage and RGC demise (Baudouin et al., 2021). Key cytokines that regulate the inflammatory response could potentially mitigate optic nerve damage and prevent the loss of RGCs linked to glaucoma (Adornetto et al., 2019). According to our meta-analysis findings, resveratrol demonstrates the ability to diminish inflammatory cytokines like iNOS, COX-2, IL-6, and IL-1β in a model of glaucoma-related retinal injury. Inhibiting or decreasing the expression of associated inflammatory factors may attenuate RGC loss and confer a protective effect on the optic nerve in glaucoma (Neufeld, 2004; Brust et al., 2008; Song et al., 2018).

Apoptosis of RGC cells significantly contributes to the pathological alterations observed in glaucoma^1924^. By inhibiting cell apoptosis, it is possible to mitigate RGC loss and potentially salvage retinal nerve damage in glaucoma (Xia and Zhang, 2024; Chitranshi et al., 2023). Our meta-analysis findings indicate that resveratrol can increase the expression of the anti-apoptotic factor Bcl-2 while decreasing the expression of pro-apoptotic factors Bcl-2 and caspase-3. Prior research has illustrated that in models of retinal ischemia-reperfusion injury induced by elevated intraocular pressure, there is a rise in caspase-3 expression levels, leading to neuronal cell death in the retina via both exogenous and endogenous pathways (Katai and Yoshimura, 1999). Similarly, investigations have revealed a stronger expression of the pro-apoptotic Bax protein in the optic nerve axons of glaucoma patients in comparison to the anti-apoptotic Bcl-2 protein (Zalewska et al., 2004). In animal models, Bax can induce dendritic degeneration in RGCs (Risner et al., 2022) and plays a crucial role in RGC apoptosis (Libby et al., 2005). Upregulation of anti-apoptotic proteins such as Bcl-2 and downregulation of pro-apoptotic proteins such as Bax can exert a protective effect on the optic nerve (Phatak et al., 2016; Maes et al., 2017).

In conclusion, our study indicates that resveratrol may increase the expression of SIRT1 protein, decrease pro-inflammatory cytokine levels, enhance anti-apoptotic factors, and suppress pro-apoptotic factors. Nonetheless, additional research is warranted to elucidate whether resveratrol is involved in the comprehensive regulation of anti-inflammatory responses, cell death mechanisms, and other pathways.

### 4.3 Limitations

Due to inherent methodological disparities, it is essential to exercise caution when extrapolating research findings from animal studies to human diseases. Although we strive to mitigate bias and enhance research accuracy by amalgamating data from multiple studies, it is important to recognize the unavoidable limitations that may impact the reliability of our results. Primarily, the variances in the animal models we incorporated possess discrepancies in replicating the extent of glaucoma-related retinal damage realistically. The absence of common clinical comorbidities like aging and diabetes could constrain the generalizability of our findings. Moreover, in instances where original research data were unattainable, a collaborative approach involving two researchers was adopted to extract data using graphic processing tools, subsequently calculating the average value for meta-analysis. However, it is important to acknowledge that this methodology may introduce inherent measurement biases. Additionally, the studies we integrated exhibited notable heterogeneity. Despite our efforts to explore potential sources of heterogeneity through sensitivity and subgroup analyses, differences in experimental design and research quality persist. Thus, for future investigations, the selection of animal models more reflective of clinical scenarios, stringent experimental protocols, and standardized methodologies are imperative to enhance research quality and facilitate clinical translation. Significantly, although we performed a basic dose-response analysis, more comprehensive pharmacological investigations are essential for the development of resveratrol as a viable drug, given the current gaps in pharmacokinetic and pharmacodynamic data.

## 5 Conclusion

Resveratrol has demonstrated protective effects on RGCs, retarding retinal thinning, and enhancing visual function in animal models of glaucoma and retinal injury. The protective mechanisms of resveratrol are likely linked to its activation of SIRT1, anti-inflammatory properties, and anti-apoptotic effects. Thus, resveratrol holds promise as a potential therapeutic agent for shielding against glaucoma-related retinal damage. However, further robust evidence is necessary in the future to facilitate its clinical translation and application.

## Data Availability

The datasets presented in this study can be found in online repositories. The names of the repository/repositories and accession number(s) can be found in the article/Supplementary Material.

## Abbreviations

RGC, retinal ganglion cell; SIRT1, silent information regulator factor 1; Brn3a, transcription factor Brn3 protein; ERG, electroretinography; IOP, intraocular pressure.
