# Supplementary material for: Protective effect of resveratrol on retinal damage in glaucoma: a systematic review and meta-analysis of preclinical studies
Source: Front Pharmacol. 2025 Jan 15;15:1521188. doi: 10.3389/fphar.2024.1521188 (PMC11774946; doi:10.3389/fphar.2024.1521188)
Supplement: Supplementary file 1 [file DataSheet1.docx]

**Supplementary materials**

**Supplementary Table 1**

Literature Retrieval Strategy for Resveratrol Treatment of Glaucoma Optic Nerve Injury

| PubMed Search Strategy | |
| --- | --- |
| #1 | Resveratrol [Mesh] |
| #2 | 3,4',5-Stilbenetriol [Title/Abstract] |
| #3 | 3,5,4'-Trihydroxystilbene [Title/Abstract] |
| #4 | 3,4',5-Trihydroxystilbene [Title/Abstract] |
| #5 | Trans-Resveratrol [Title/Abstract] |
| #6 | Trans Resveratrol [Title/Abstract] |
| #7 | Resveratrol-3-sulfate [Title/Abstract] |
| #8 | Resveratrol 3 sulfate [Title/Abstract] |
| #9 | SRT 501[Title/Abstract] |
| #10 | SRT-501 [Title/Abstract] |
| #11 | SRT501 [Title/Abstract] |
| #12 | Cis-Resveratrol [Title/Abstract] |
| #13 | Cis Resveratrol [Title/Abstract] |
| #14 | Resveratrol, (Z)- [Title/Abstract] |
| #15 | Trans-Resveratrol-3-O-sulfate [Title/Abstract] |
| #16 | Trans Resveratrol 3 O sulfate [Title/Abstract] |
| #17 | #1 OR #2 OR #3 OR #4 OR #5 OR #6 OR #7 OR #8 OR #9 OR #10 OR #11 OR #12 OR #13 OR #14 OR #15 OR #16 |
| #18 | Glaucoma [Mesh] |
| #19 | Glaucomas [Title/Abstract] |
| #20 | Ocular Hypertension [Mesh] |
| #21 | Hypertension, Ocular [Title/Abstract] |
| #22 | Hypertensions, Ocular [Title/Abstract] |
| #23 | Ocular Hypertensions [Title/Abstract] |
| #24 | Glaucoma, Suspect [Title/Abstract] |
| #25 | Glaucomas, Suspect [Title/Abstract] |
| #26 | Suspect Glaucoma [Title/Abstract] |
| #27 | Suspect Glaucomas [Title/Abstract] |
| #28 | #18 OR #19 OR #20 OR #21 OR #22 OR #23 OR #24 OR #25 OR #26 OR #27 |
| #29 | Retina [Mesh] |
| #30 | Ora Serrata [Title/Abstract] |
| #31 | Retinal Neurons[Mesh] |
| #32 | Neuron, Retinal [Title/Abstract] |
| #33 | Neurons, Retinal [Title/Abstract] |
| #34 | Retinal Neuron [Title/Abstract] |
| #35 | #29 OR #30 OR #31 OR #32 OR #33 OR #34 |
| #36 | #17 AND #34 |
| Web of science search strategy | |
| #1 | (((((((((((((((TS=(Resveratrol)) OR TS=(3,4',5-Stilbenetriol)) OR TS=( 3,5,4'-Trihydroxystilbene)) OR TS=(3,4',5-Trihydroxystilbene)) OR TS=(Trans-Resveratrol)) OR TS=(Trans Resveratrol)) OR TS=(Resveratrol-3-sulfate)) OR TS=(Resveratrol 3 sulfate)) OR TS=(SRT 501)) OR TS=( SRT-501)) OR TS=(SRT501)) OR TS=( Cis-Resveratrol)) OR TS=(Cis Resveratrol)) OR TS=(Resveratrol, (Z)-)) OR TS=(Trans-Resveratrol-3-O-sulfate)) OR TS=(Trans Resveratrol 3 O sulfate) |
| #2 | (((((((((((((((TS=(Glaucoma)) OR TS=(Glaucomas)) OR TS=(Ocular Hypertension)) OR TS=(Hypertension, Ocular)) OR TS=(Hypertensions, Ocular)) OR TS=(Ocular Hypertensions)) OR TS=(Glaucoma, Suspect)) OR TS=(Glaucomas, Suspect)) OR TS=( Suspect Glaucoma)) OR TS=(Suspect Glaucomas)) OR TS=(Retina)) OR TS=(Ora Serrata)) OR TS=(Retinal Neurons)) OR TS=(Neuron, Retinal)) OR TS=(Neurons, Retinal)) OR TS=(Retinal Neuron) |
| #3 | #1 AND #2 |
| Embase search strategy | |
| #1 | 'resveratrol'/exp |
| #2 | '3, 4`, 5 stilbenetriol' OR '3, 4`, 5 trihydroxystilbene' OR '5 (4 hydroxystyryl) benzene 1, 3 diol' OR 'srt 501' OR 'srt501' OR 'trans resveratrol' OR 'trans-resveratrol' OR 'resveratrol':ab, ti |
| #3 | #1 OR #2 |
| #4 | 'glaucoma'/exp |
| #5 | 'chronic glaucoma' OR 'glaucoma':ab, ti |
| #6 | #4 OR #5 |
| #7 | 'intraocular hypertension'/exp |
| #8 | 'eye hypertension' OR 'hypertension, eye' OR 'hypertension, ocular' OR 'ocular hypertension' OR 'intraocular hypertension':ab, ti |
| #9 | #7 OR #8 |
| #10 | 'retina'/exp |
| #11 | 'retina profile' OR 'retina threshold' OR 'retina':ab, ti |
| #12 | #10 OR #11 |
| #13 | 'retina nerve cell'/exp |
| #14 | 'nerve cell, retina' OR 'neural retina cell' OR 'retina neuron' OR 'retinal nerve cell' OR 'retinal neuron' OR 'retinal neurons' OR 'retina nerve cell':ab, ti |
| #15 | #13 OR #14 |
| #16 | #6 OR #9 OR #12 OR #15 |
| #17 | #3 AND #16 |
| CBM search strategy | |
| #1 | Resveratrol [Mesh] |
| #2 | Trans Resveratrol |
| #3 | Resveratrol 3 sulfate |
| #4 | Cis Resveratrol |
| #5 | Trans Resveratrol 3 O sulfate |
| #6 | #1 OR #2 OR #3 OR #4 OR #5 |
| #7 | Glaucoma [Mesh] |
| #8 | Ocular Hypertension [Mesh] |
| #9 | Suspect Glaucoma |
| #10 | Retina [Mesh] |
| #11 | Retinal Neuron |
| #12 | #7 OR #8 OR #9 OR#10 OR #11 |
| #13 | #6 AND #12 |
| CNKI search strategy | |
| #1 | Resveratrol (subjects) OR Trans Resveratrol (subjects) OR Resveratrol 3 sulfate (subjects) OR Trans Resveratrol 3 O sulfate (subjects)OR Cis Resveratrol (subjects) |
| #2 | Glaucoma (subjects) OR Ocular Hypertension (subjects) OR Retina(subjects) OR Retinal Neuron(subjects) |
| #3 | #1 AND #2 |
| WF search strategy | |
| #1 | Subjects:(“Resveratrol” OR “Trans Resveratrol” OR “Resveratrol 3 sulfate” OR “Trans Resveratrol 3 O sulfate” OR “Cis Resveratrol”) AND subjects(“Glaucoma” OR “Ocular Hypertension” OR “Retina” OR “Retinal Neuron”) |
| VIP search strategy | |
| #1 | K=(Resveratrol OR Trans Resveratrol OR Resveratrol 3 sulfate OR Trans Resveratrol 3 O sulfate OR Cis Resveratrol) AND K=(Glaucoma OR Ocular Hypertension OR Retina OR Retinal Neuron) |

**Supplementary Table 2** Risk of Bias Summary

| Author(year) | A | B | C | D | E | F | G | H | I | J | Total |
| --- | --- | --- | --- | --- | --- | --- | --- | --- | --- | --- | --- |
| Pirhan et al.(2015) | ? | + | ? | + | ? | + | + | + | + | + | 7 |
| Seong et al. (2017) | ? | + | ? | + | ? | + | ? | + | + | + | 6 |
| Chronopoulos et al. (2023) | ? | + | ? | ? | ? | + | ? | + | + | + | 5 |
| Luo et al. (2018) | ? | + | ? | ? | ? | + | ? | + | + | + | 5 |
| Pang et al. (2020) | ? | + | ? | ? | ? | + | ? | + | + | + | 5 |
| Cao et al. (2020) | ? | + | ? | ? | ? | + | ? | + | + | + | 5 |
| Xie et al. (2023) | ? | + | ? | + | ? | + | ? | + | + | + | 6 |
| Vin et al. (2013) | ? | + | ? | ? | ? | + | ? | + | + | + | 5 |
| Feng et al. (2024) | ? | + | ? | ? | ? | + | ? | + | + | + | 5 |
| Wu et al. (2020) | ? | + | ? | ? | ? | + | ? | + | + | + | 5 |
| Xia et al. (2020) | ? | ? | ? | ? | ? | + | ? | + | + | + | 4 |
| Deng et al. (2020) | ? | + | ? | ? | ? | + | ? | + | + | + | 5 |
| Zhang et al. (2018) | ? | + | ? | ? | ? | + | ? | + | + | + | 5 |
| Ji et al. (2024) | ? | + | ? | + | ? | + | ? | + | + | + | 5 |
| Prasetya et al. (2023) | + | + | ? | ? | ? | + | ? | + | + | + | 6 |
| Zhao et al. (2022) | ? | + | ? | + | ? | + | ? | + | + | + | 6 |
| Seong et al. (2022) | ? | + | ? | + | ? | + | ? | + | + | + | 6 |
| Su et al. (2018) | ? | + | ? | ? | ? | + | ? | + | + | + | 5 |
| Liu et al. (2013) | ? | + | ? | ? | ? | + | ? | + | + | + | 5 |
| Chen et al. (2018) | ? | + | ? | ? | ? | + | ? | + | + | + | 5 |
| Luo et al. (2020) | ? | + | ? | + | ? | + | ? | + | + | + | 6 |
| Xiong et al. (2021) | ? | ? | ? | ? | ? | + | ? | + | + | + | 4 |
| Li et al. (2012) | ? | ? | ? | + | ? | + | ? | + | + | + | 5 |
| Ji et al. (2016) | ? | + | ? | ? | ? | + | ? | + | + | + | 5 |
| Ji et al. (2022) | ? | + | ? | ? | ? | + | ? | + | + | + | 5 |
| Zhou et al. (2016) | ? | + | ? | + | ? | + | ? | + | + | + | 6 |
| Qi et al. (2020) | + | + | ? | + | ? | + | ? | + | + | + | 7 |
| He et al. (2021) | + | ? | ? | + | ? | + | ? | + | + | + | 5 |
| Shamsher et al. (2022) | ？ | + | ? | ？ | ? | + | ? | + | + | + | 5 |
| Zhu et al. (2018) | + | ？ | ? | ？ | ? | + | ? | + | + | + | 5 |

1. Sequence generation. (B) Baseline characteristics. (C) Allocation concealment. (D) Random housing. (E) Blinding of experimentalists. (F) Random outcome assessment. (G) Blinding of outcome assessors. (H) Incomplete outcome data. (I) Selective outcome reporting. (J) Other sources of bias. +: indicates low risk; -: indicates high risk; ?: indicates unclear risk.

**Supplementary Table 3** The subgroup analyses of Survival status of RGC, Death status of RGC, Brn3a ，and Retinal thickness.

| **Outcome** | **Subgroup** |  | **No. studies** | **SMD [95% CI]** | ***I*^2^** |
| --- | --- | --- | --- | --- | --- |
| **Survival status of RGC** | Modeling method | **Anterior chamber perfusion method** | 14 | 4.40 [3.13, 5.67] | **72.1%** |
|  |  | **Cauterization of scleral vein method** | 1 | 4.75 [2.97, 6.53] | **-** |
|  |  | **Injecting specific materials or drugs into the anterior chamber** | 4 | 4.07 [1.41, 6.74] | **89.1%** |
|  | Species | **Sprague-Dawley rats** | 6 | 5.25 [2.68, 7.81] | **84.2%** |
|  |  | **Wistar rats** | 3 | 4.97 [3.13, 6.83] | **63.2%** |
|  |  | **C57BL/6J mice** | 9 | 3.71 [2.26, 5.15] | **71.8%** |
|  |  | Agouti rats | 1 | 3.75 [1.53, 5.96] | **-** |
|  | Administration method | **Intraperitoneal injection** | 12 | 5.02[4.10, 5.95] | **36.9%** |
|  |  | **Intravitreous injection** | 3 | 4.52 [2.28, 6.75] | **47.7%** |
|  |  | **Gavage or peros** | 3 | 1.67 [-0.03, 3.38] | **79.3%** |
|  |  | **Eye drops** | 1 | 3.75 [1.53, 5.96] | **-** |
|  | Administered dose | 0.2-0.5 nmol (Injection) | 2 | 3.44 [1.77, 5.15] | **0%** |
|  |  | 0-150mg/kg (Injection) | 8 | 4.60 [3.36, 5.85] | **40.9%** |
|  |  | 151-300mg/kg (Injection) | 3 | 6.34 [4.60,8.09] | **0%** |
|  |  | ＞300 mg/kg (Injection) | 2 | 5.85 [3.79,7.36] | **40.7%** |
|  |  | 20-30 mg/kg/d(Oral) | 3 | 1.67 [-0.03, 3.38] | **79.3%** |
|  |  | NM (Eye drops) | 1 | 3.75 [1.53, 5.96] | **-** |
|  | **Administration time** | Before molding | 4 | 2.84 [0.74, 4.95] | **77.0%** |
|  |  | Before molding until a certain period of time after molding | 11 | 4.78 [3.68, 5.89] | **42.2%** |
|  |  | After molding | 4 | 4.32 [1.69, 6.94] | **89.9%** |
| **Death status of RGC** | Modeling method | **Anterior chamber perfusion method** | 2 | -2.27[-3.77, -0.77] | **47.6%** |
|  |  | **Injecting specific materials or drugs into the anterior chamber** | 6 | -5.07[-7.46, -2.68] | **89.6%** |
|  | Species | **Sprague-Dawley rats** | 3 | -4.39[-5.99, -2.79] | **60.2%** |
|  |  | **Wistar rats** | 2 | -6.17[-7.58 -4.76] | **0%** |
|  |  | **C57BL/6J mice** | 2 | -2.18[-3.48, -0.87] | **34.6%** |
|  | Administration method | Agouti rats | 1 | -1.43[-2.85, -0.00] | **-** |
|  |  | **Intraperitoneal injection** | 3 | -4.63[-7.99, -1.26] | **91%** |
|  |  | **Intravitreous injection** | 2 | -3.08[-4.32, -1.83] | **0%** |
|  |  | **Gavage or peros** | 2 | -5.03[-6.66, -3.40] | **42.6%** |
|  |  | **Eye drops** | 1 | -1.43[-2.85, -0.00] | **-** |
|  | Administered dose | 0.2-0.5 nmol (Injection) | 2 | -3.08 [-4.32, -1.83] | **0%** |
|  |  | 0-150mg/kg (Injection) | 2 | -5.70 [-6.84, -4.57] | **0%** |
|  |  | ＞300 mg/kg (Injection) | 2 | -4.13 [-9.19, 0.93] | **93.8%** |
|  |  | 20-30 mg/kg/d(Oral) | 1 | -4.00 [-6.09, -1.92] | - |
|  |  | NM (Eye drops) | 1 | -1.43 [-2.85, -0.00] | - |
|  | **Administration time** | Before molding | 1 | -3.18[-4.98, -1.39] | - |
|  |  | Before molding until a certain period of time after molding | 1 | -1.43[-2.85, -0.00] | - |
|  |  | After molding | 6 | -4.41[-6.10, -2.71] | **83.1%** |
| **Brn3a** | Modeling method | **Anterior chamber perfusion method** | 6 | 4.03 [1.75, 6.32] | **84.8%** |
|  |  | **Injecting specific materials or drugs into the anterior chamber** | 1 | 1.94 [0.52, 3.35] | - |
|  | Species | **Sprague-Dawley rats** | 2 | 3.84 [2.01, 5.67] | **34.6%** |
|  |  | **C57BL/6J mice** | 5 | 3.49 [1.15, 5.84] | **85.1%** |
|  | Administration method | **Intraperitoneal injection** | 3 | 5.18 [2.17, 8.18] | **59.6%** |
|  |  | **Intravitreous injection** | 2 | 3.29 [0.31, 6.28] | **77.6%** |
|  |  | **Gavage or peros** | 2 | 2.20[−1.95, 6.34] | **92.8%** |
|  | Administered dose | 0.2-0.5 nmol (Injection) | 1 | 1.94 [0.52, 3.35] | **-** |
|  |  | 0-150mg/kg (Injection) | 2 | 7.01 [4.04, 9.98] | **0%** |
|  |  | 151-300mg/kg (Injection) | 2 | 3.84[2.01, 5.67] | **34.6%** |
|  |  | 20-30 mg/kg/d(Oral) | 2 | 2.20 [−1.95, 6.34] | **92.8%** |
|  | **Administration time** | Before molding | 1 | 0.15 [-1.00, 1.30] | **-** |
|  |  | Before molding until a certain period of time after molding | 5 | 4.54 [3.24, 5.84] | **24.7%** |
|  |  | After molding | 1 | 1.94 [0.52, 3.35] | **-** |
|  |  | Before ischemia up to a certain period after reperfusion. | 3 | -3.26[−4.10, −2.41] | **0%** |
| **Retinal thickness** | Modeling method | **Anterior chamber perfusion method** | 10 | 4.46 [2.58, 6.34] | **84.4%** |
|  |  | **Injecting specific materials or drugs into the anterior chamber** | 2 | 3.64 [2.01, 5.26] | **40.4%** |
|  | Species | **Sprague-Dawley rats** | 6 | 5.30 [3.88, 6.73] | **23.1%** |
|  |  | **Wistar rats** | 1 | 3.06 [3.88, 6.73] | **-** |
|  |  | **C57BL/6J mice** | 5 | 3.26 [0.94, 5.57] | **86.5%** |
|  | Administration method | **Intraperitoneal injection** | 8 | 4.34 [2.50, 6.17] | **80.7%** |
|  |  | **Intravitreous injection** | 2 | 6.19 [4.00, 8.38] | **0%** |
|  |  | **Gavage or peros** | 2 | 2.52 [-1.70, 6.78] | **90.1%** |
|  | Administered dose | 0.2-0.5 nmol (Injection) | 2 | 6.19 [4.00, 8.38] | **0%** |
|  |  | 0-150mg/kg (Injection) | 6 | 4.30 [2.08, 6.52] | **84.4%** |
|  |  | 151-300mg/kg (Injection) | 1 | 6.87 [2.74, 10.99] | **-** |
|  |  | ＞300 mg/kg (Injection) | 1 | 3.11 [0.87, 5.35] | **-** |
|  |  | 20-30 mg/kg/d(Oral) | 2 | 2.54 [-1.70, 6.78] | **90.1%** |
|  | **Administration time** | Before molding | 4 | 2.87 [0.42, 5.35] | **86.9%** |
|  |  | Before molding until a certain period of time after molding | 6 | 5.34 [3.88, 6.80] | **22.8%** |
|  |  | After molding | 2 | 3.64 [2.01, 5.26] | **40.4%** |

**Supplementary Figure 1** The results of the publication bias

**
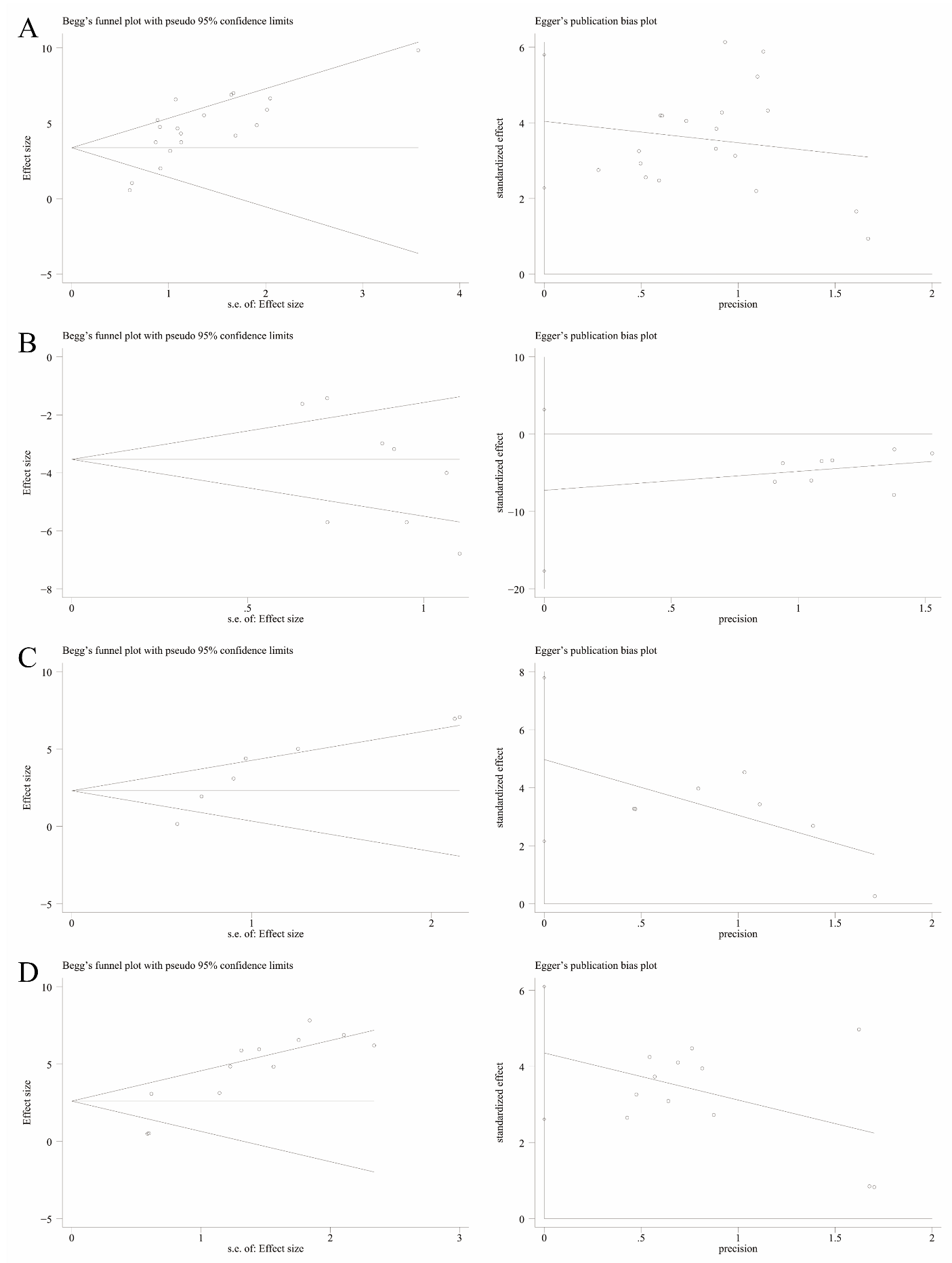
**

Begg’s and Egger’s publication bias plot for (A)Survival status of RGC, (B) Death status of RGC, (C) Brn3a,and (D) Retinal thickness.
